# Schieffelin & Co’s Extracts

**Published:** 1850-07

**Authors:** 


*Schieffelin & Co's Extracts.*—We have examined some of the extracts prepared by Schieffelin & Co., of New York, and we do not hesitate to pronounce them, in our judgment, superior to any that have, as yet, fallen under our notice. So large a proportion of the extracts heretofore sold by Druggists and Apothecaries has been found to be worthless, that they have of late fallen into comparative disuse. The introduction of better articles is certainly a great desideratum.

